# The association between methylation levels of targeted genes and albuminuria in patients with early diabetic kidney disease

**DOI:** 10.1080/0886022X.2017.1358180

**Published:** 2017-08-14

**Authors:** Ozgur Aldemir, Faruk Turgut, Cumali Gokce

**Affiliations:** a Department of Medical Genetics, School of Medicine, Mustafa Kemal University, Hatay, Turkey;; b Department of Internal Medicine, Nephrology, Mustafa Kemal University, School of Medicine, Hatay, Turkey;; c Department of Internal Medicine, Endocrinology, Mustafa Kemal University, School of Medicine, Hatay, Turkey

**Keywords:** Type 2 diabetes, diabetic nephropathy, microalbuminuria, TIMP-2, AKR1B1, methylation

## Abstract

**Objective:** The incidence of diabetes and its complications are greatly increasing world-wide. Diabeticnephropathy (DN) is the main cause of end-stage renal disease and is associated with high morbidity and mortality. It is important to predict patients with high risk for DN in the early stage. We selected the genes which have an important role on diabetic kidney disease. We aimed to investigate the association between DNA methylation levels of targeted genes and albuminuria in patients with early DN.

**Methods:** We collected the clinical data of patients with type 2 diabetes mellitus. We measured spot urine albumin creatinine ratio to calculate albuminuria level. We divided patients into two groups based on albumin excretion as patients with (*n* = 69) and without DN (*n* = 27). We performed methylation profiling after bisulfite conversion by pyrosequencing method. The mean value of percent methylation level of each gene was calculated.

**Results:** We compared targeted genes (TIMP-2, AKR1B1, MMP-2, MMP-9, MYL9, SCL2A4, SCL2A1, SCL4A3) methylation levels and albuminuria. We found significant negative correlation between TIMP-2 and AKR1B1 gene methylation levels and albuminuria levels.

**Conclusions:** The present study provided evidence that hypomethylation of TIMP-2 and AKR1B1 genes can be associated with albuminuria in patients with early DN. We may speculate that the hypomethylation of TIMP-2 and AKR1B1 genes may be an early surrogate marker of DN.

## 1. Introduction

The incidence and prevalence of diabetes mellitus (DM) have grown significantly throughout the world as in Turkey [1,2]. In the same line, diabetic microvascular and macrovascular complications including atherosclerotic cardio-vascular disease and diabetic nephropathy has become the major determinants of morbidity, mortality and economical costs in case of the disorder [1]. Diabetic nephropathy (DN) is a serious microvascular complication of type 2 diabetes mellitus (T2D) and it is the most common cause of end-stage renal disease. The treatment of DN has been more expensive and difficult to be successful in this process. In addition, DN has serious economic burden in many developed countries across the world.

DN is not only associated with chronic kidney disease (CKD) but in also increased risk of cardiovascular disease [3]. Management of CKD is costly and prevention or slowing the progression of CKD is cost-effective [4]. Some of the adverse outcomes of DN can be prevented or delayed by some assays or early detection. The pathogenesis of DN is complex and so many factors contribute to its pathogenesis including genetic and environmental factors. Epigenetic factors are involved with the complex interplay between genes and the environment. Epigenetic factors mainly include DNA methylation changes that has been considered to be involved in this pathogenesis [5]. Epigenetics refers to heritable changes in gene expression and ensuing phenotypes that occur without changes in the DNA sequence. Epigenetic changes include DNA methylation, histone post-translational modifications (PTMs) in chromatin and non-coding RNAs (ncRNAs), all of which can affect gene expression individually or co-operatively and modulate disease states [6]. It is hypothesized that epigenetic mechanisms may have a role in the pathogenesis of DN, possibly promoting genetic tendencies for T2D-associated complications [5]. Microalbuminuria is recognized as an important marker of early-stage DN to prevent the progression of renal dysfunction [7]. We speculated whether there is a predictive epigenetic biomarker in the early stage of DN. Therefore, in the present study, we aimed to investigate the association between the methylation levels of targeted genes and albuminuria levels in T2D patients with and without DN. We hypothesized to predict patients with high risk for DN in the early stage.

## 2. Material and methods

### 2.1. Sociodemographic, clinical data and biochemical analyzes

We collected clinical data and sociodemographic data of patients with T2D. Hypertension was defined by systolic blood pressure ≥140 mmHg and/or diastolic blood pressure ≥90 mmHg. Participants currently using antihypertensive medications were also classified as positive for hypertension. Individuals complicated with other kidney diseases, such as chronic glomerulonephritis and interstitial nephritis, were excluded from the study. Estimated glomerular filtration rate (eGFR) was calculated using the formula reported by the modification of diet in renal disease (MDRD) study group [8].

Early morning spot urine specimens were collected from the participants and no albuminuria testing was performed on visibly hematuria or hemoglobin dipstick-positive specimens. Urinary albumin was measured by fluorescent immunoassay and urinary creatinine by the kinetic alkaline picrate (modified Jaffé, Autoanalyzer) method. A urinary albumin-to-creatinine ratio is calculated and the urinary albumin excretion is presented as the albumin-to-creatinine ratio (ACR; mg/g creatinine). DN was staged according to analysis of a spot urine sample as follows: normoalbuminuria, ACR <30 mg/g creatinine; microalbuminuria, 30 ≤ ACR <300 mg/g creatinine; macroalbuminuria, ACR ≥300 mg/g creatinine. We excluded patients with proteinuria higher than 1000 mg/g [9].

### 2.2. Bisulfite treatment and methylation analyzes in pyrosequencing

We extracted DNA samples from peripheral blood using Qiagen EZ1 Blood kit. We analyzed the DNA methylation levels of eight genes (TIMP-2, AKR1B1, MYL9, MMP-2, MMP-9, SCL2A1, SCL2A4 and SCL4A3) as a case control study (48 T2D patients with DN and 48 T2D patients without DN) with standard protocol of bisulfite and pyrosequencing. First of all, DNA was treated with sodium bisulfite using EpiTect bisulphite kit (Qiagen) and clean-up of bisulfite-converted DNA was made. PCR amplification was achieved using PyroMark CpG assay (Qiagen ID: 6508,6517,6513,10398,4318,7077,231) and PyroMark Gold Q24 Reagents kits (Qiagen) in a PyroMark Q24 system (BiotageAb, Uppsala, Sweden). PyroMark PCR master mix includes Hotstar Taq DNA polymerase and optimized PyroMark reaction buffer containing 3nMCl2 and dNTPs 10X CoralLoad Concentrate, 5x Q-Solution, 25 nM MgCl_2_ and RNase free water. DNA methylation levels of the eight genes CpG island sites were discovered by using PyroMark Gold 24 reagent kit (Qiagen) and a PyroMark Q24 ID Pyrosequencing system (Biotage, Uppsala, Sweden). The unmethylated and unconverted DNA samples (Qiagen) were used for control of conversion efficiency in bisulfite treatment and accuracy in methylation analyzes. PyroQ-CpG software (Biotage) was used for methylation data analysis.

### 2.3. Statistical analyses

All statistical analyses were performed using the SPSS program, version 21.0 (SPSS Inc., Chicago, IL). Unless otherwise stated, values are expressed as means ± SD. To compare the differences between groups, Student’s *t* test or Mann–Whitney *U* test were used for continuous variables as appropriate based on distribution. Chi-square test was used for categorical variables. A bivariate analysis was performed to investigate the association between gene methylation levels and albuminuria level. The non-parametric Spearman rho coefficient of correlation was used to assess correlations between variables with non-normal distribution. *p* < .05 was considered statistically significant. We did not calculate statistical significane between eGFR and methylation level of selected genes.

## 3. Results

Methylation status of those genes was determined in a total of 96 diabetic patients. Clinical characteristics and some laboratory parameters are given in Table 1. Apart from gender all demographic characteristics were similar between groups. T2D patients with macroalbuminuria had significantly higher eGFR, which may be expected in this patient group. Based on spot urine albumin creatinine ratio, 26 patients had normoalbuminuria (14.00 ± 8.14) and 70 patients were either microalbuminuria (136.56 ± 59.52) (*n* = 59) or macroalbuminuria (628.38 ± 217.39) (*n* = 11).The biochemical and clinical parameters for all subjects are summarized in Table 1. We have shown the mean methylation levels of each gene in Table 2. The study showed that there are a large number of methylation changes in the promotor region of genes. MYL9 is hypermethylated (87%) and TIMP-2, AKR1B1, MMP-2, MMP-9, SCL2A4, SCL2A1, SCL4A3 are hypomethylated, with mean levels 8.8, 4.3, 4.1, 4.6, 6.2, 21.9 and 3.2%, respectively. The selected genes (TIMP-2, AKR1B1, MMP-2, MMP-9, MYL9, SCL2A4, SCL2A1, SCL4A3) are associated with different cellular pathways for the progression of T2D to T2D with microalbuminuria and macroalbuminuria. The mean values of methylation levels in microalbuminuria group are TIMP-2 level (8.6 ± 1.18), SLC2A1(4.11 ± 0.66), SLC2A4 (3.94 ± 0.80), MYL9 (86.80 ± 2.17), MMP2 (3.82 ± 0.84), SLC4A3 (6.18 ± 0.84), MMP9 (23.4 ± 11.12) and AKR1B1 (3.64 ± 1.64), respectively. The mean values of methylation levels in macroalbuminuria group are TIMP-2 level (8.18 ± 1.60), SLC2A1 (3.99 ± 1.01), SLC2A4 (4.03 ± 1.1), MYL9 (86.88 ± 1.93), MMP2 (4.67 ± 2.3), SLC4A3 (5.93 ± 1.08), MMP9 (25.91 ± 9.9) and AKR1B1 (3.12 ± 1.14). There was no significant changes for methylation levels of six genes (including MYL9, MMP-2, MMP-9 SCL2A1, SCL2A4 and SCL4A3) between T2D patients with and without DN. However, there was a significant negative correlation with the mean value of TIMP-2 and AKR1B1 methylation level and albuminuria (Table 2). There was no association between clinical, sociodemografic data, laboratory parameters (Hb, HbA1c, eGFR, CRP, Total cholesterol, LDL, HDL, TG) shown in Table 1 and the mean value of targeted genes methylation levels.

**Table 1.** The clinical, socio demographic data and the ratio of methylation levels.

Group	All T2D (*n* = 96)	T2-DN (*n* = 26)	T2 + DN (*n* = 70)	*p*-Value
Gender (female/male)	49/47	18/8	31/39	.03
Age (years)	54.9 ± 9.1	54 ± 9	55 ± 8	.57
BMI (kg/m^2^)	30.8 ± 4.7	31.5 ± 6.0	29.9 ± 5.2	.97
SBP (mmHg)	138 ± 15	135 ± 17	140 ± 14	.10
DBP (mmHg)	81 ± 10	79 ± 7	83 ± 10	.10
HbA1C (%)	8.6 ± 2.1	8.5 ± 2.2	9.1 ± 1.5	.31
eGFR (ml/min/1.73 m^2^)	98.1 ± 19.3	92.4 ± 18.9	100.5 ± 25.2	.03
Hb (g/dL)	13.2 ± 1.6	13.3 ± 1.3	13.1 ± 2.1	.92
CRP (mg/L)	5.8 ± 6.4	4.8 ± 3.5	6.5 ± 6.9	.31
Albuminuria	159 ± 196	14 ± 8.1	213 ± 205	.0001
Total Cholesterol (mg/dl)	203 ± 44	199 ± 54	205 ± 40	.70
HDL Cholesterol (mg/dl)	39 ± 12	40 ± 12	39 ± 12	.48
LDL Cholesterol (mg/dl)	128 ± 38	131 ± 17	127 ± 39	.73
Triglyceride (mg/dl)	186 ± 106	174 ± 89	190 ± 112	.44

Data are expressed as means ± SD; T2D: type 2 diabetes; DN: diabetic nephropathy; BMI: body mass index; SBP and DBP: systolic and diastolic blood pressures; HbA1c: glycated hemoglobin; eGFR: estimated glomerular filtration rate; Hb: Hemoglobin; CRP: C-reactive protein; *p* values were from tests of all T2D-DN (Type 2 Diabetes without DN) vs. T2D + DN (Type 2 Diabetes with DN).

**Table 2.** The spearman correlation results between proteinuria level and the methylation levels of target genes.

Genes	*R* value	*p* value
TIMP-2	−0.253	.013
AKR1B1	−0.228	.026
SLC2A1	−0.098	.342
SLC2A4	−0.082	.429
MYL9	−0.004	.969
MMP-2	0.075	.470
MMP-9	0.071	.494
SLC4A3	−0.168	.101

## 4. Discussion

**Figure 1.**
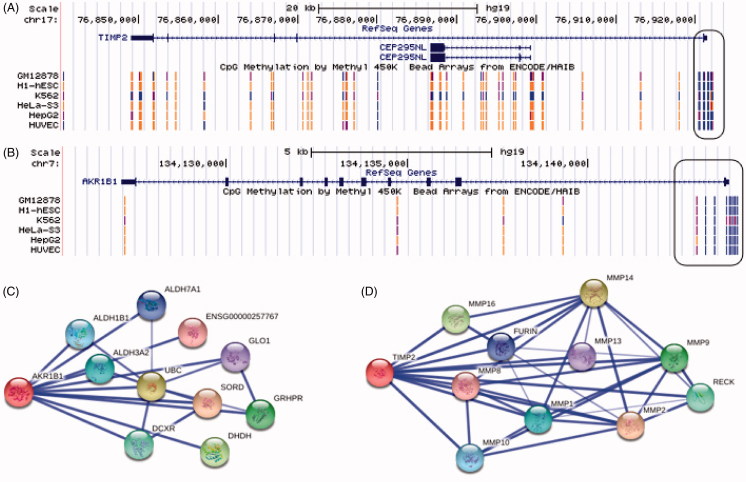
(A and B): Display of TIMP-2 and AKR1B1 gene structure and methylation pattern. The promotor region methylation pattern at some cell line as showed in hypermethylation is represented by blue dots, however the present study provided evidence that DNA hypomethylation of TIMP-2 and AKR1B1 gene promotor (Data were collected from http://genome.ucsc.edu/). (C): AKR1B1 and interacted genes. Known to act on SORD (sorbitol dehydrogenase), UBC (ubiquitin C), DHDH (dihydrodiol dehydrogenase), GRHPR (glyoxylate reductase/hydroxypyruvate reductase), DCXR (dicarbonyl/L-xylulose reductase), ALDH3A2 (aldehyde dehydrogenase 3 family), ALDH1B1 (aldehyde dehydrogenase 1 family), ALDH7A1 (aldehyde dehydrogenase 7 family), GLO1 (glyoxalase I) and ENSG00000257767 (Uncharacterized protein). (D): TIMP-2 and interacted genes. Known to act on MMP1 (matrix metallopeptidase 1), MMP2 (matrix metallopeptidase 2), MMP4 (matrix metallopeptidase 4), MMP10 (matrix metallopeptidase 10), MMP16 (matrix metallopeptidase 16), MMP9 (matrix metallopeptidase 9), MMP13 (matrix metallopeptidase 13), MMP8 (matrix metallopeptidase 8), RECK (reversion-inducing-cysteine-rich protein with kazal motifs) and FURIN (furin).

In the study, we targeted the key genes from Sapienza et al. [10] study which take part in the important biochemical pathways such as polyol pathway and increased extracellular matrix (ECM) protein. We found a significant correlation between the methylation levels of TIMP-2, AKR1B1 genes with microalbuminuria, macroalbuminuria and in T2D patients with and without DN. Up-to-date, genome-wide association studies (GWASs) have been identifying a number of genetic variations in diabetes for DN. There are a few meta-analysis about the genetic basis of DN in the literature. According to the literature, there are different genetic variants associated with DN that explored the association between the genetic variants within the genes involved in inflammatory cytokines, angiogenesis pathways in DN. Nazir et al. (2014) [11] have showed that 11 genetic variants were significantly associated with DN, and there was strong relationship between inflammation and the development and progression of DN. Their results support for a role in the inflammatory cytokines and angiogenesis pathways in the pathogenesis of DN. The present study demonstrated that there are a large number of methylation levels of AKR1B1 and TIMP-2 genes (shown in Figure 1). Yamashita et al. [12] performed AKR1B1 gene expression in non-diabetics, diabetics with and without DN. They found significant differences in AKR1B1/beta-actin mRNA ratios between both diabetics and non-diabetics with kidney disease and controls. They concluded that the degree of AKR1B1 gene expression modulates the risk for nephropathy in type 1 DM. In the present study, we speculated that the hypomethylation level of TIMP-2 and AKR1B1 take part in the ECM degradation and polyol pathway. It may be a new predictive biomarker and a new molecular target to surrogate early and for treatment of DN. Several mechanisms have been proposed to explain how AKR1B1 (aldose reductase 1 beta 1) gene activity leads to hyperglycemia-induced lesions in different tissues, such as at high concentrations in the seminal vesicles, lens, retina, renal medulla and sciatic nerve. AKR1B1 is associated with diabetic neuropathy and diabetic autonomic neuropathy [13,14]. The present study found for the first time a negative correlation between AKR1B1 methylation level and albuminuria in DN. We speculate that AKR1B1 enzyme activity may be associated with diabetic nephropathy.

TIMP (tissue inhibitor of metalloprotease) proteins encoded by this gene family are natural inhibitors of the matrix metalloproteinases, a group of peptidases involved in degradation of the ECM. In addition to an inhibitory role against metalloproteinases, the encoded protein has a unique role among TIMP family members in its ability to directly suppress the proliferation of endothelial cells and they were related in an important role in the pathogenesis of DN. The present study showed that TIMP-2 hypomethylation in DN patients may lead to ECM degradation, therefore TIMP-2 methylation level is associated with increased fibronectin expression and ECM accumulation. TIMPs are considered to maintain homeostasis between production and degradation of ECM in the renal glomeruli. Confined imbalance between MMP and TIMP activity was demonstrated to be involved in diabetic renal remodeling. High glucose levels decreased expression of MMPs and increased expression of TIMPs by decreasing plasmin availability and reducing expression of membrane-bound MMPs and up-regulated TGF-sz, which in turn could alter MMP promoter activity [15]. Further, the increase of MMP-9 concentration was shown to precede and predict microalbuminuria in patients with T2D [16,17]. Sapienza et al. [10] performed DNA methylation levels at more than 27,000 CpG sites in more over 14,000 genes of diabetes patients with or without end-stage renal disease to assess the possibility that epigenetic factors play a role in DN. In their GWAS study, they identified 187 genes that are differentially methylated between the two groups on at least two CpG sites in each gene in DNA samples. The epigenetic profiling study showed us that there are only 39 gene methylation levels related with DN [18]. The present study showed that there are a large number of methylation changes in all targeted genes such as TIMP-2, AKR1B1, MMP-2, MMP-9, MYL9, SCL2A4, SCL2A1, SCL4A3. We found an association between the hypomethylation of TIMP-2 gene, AKR1B1 gene and albuminuria in early stages of DN.

There are some limitations in the present study. First, the number of studied patients is relatively small. Second, a healthy control group was not included in the present study. But, we only aimed to investigate those diabetic patients with albuminuria and without albuminuria. Third, we measured spot urine albumin/creatinine ratio, which is not gold standard to define 24 h albuminuria. However, studies validate that early morning urine analysis is as accurate as 24 h urine collection. Finally, there was a gender difference between groups, which may be another limitation in the present study.

In conclusion, we not only provided insights into the pathogenesis of diabetic nephropathy but also discovered potential biomarkers for diagnosis or detection and treatment. Further methylation studies with large samples are needed to compare with non-diabetic/pre-diabetic cases to T2D with DN. It should be kept in mind that the methylation level of targeted genes are possibly presented to understanding the pathophysiology of DN and this can be used to discover new molecular treatment approach.
